# The role of collective efficacy in long‐term condition management: A metasynthesis

**DOI:** 10.1111/hsc.12779

**Published:** 2019-06-24

**Authors:** Ivaylo Vassilev, Rebecca Band, Anne Kennedy, Elizabeth James, Anne Rogers

**Affiliations:** ^1^ NIHR Collaboration for Leadership in Applied Health Research (CLAHRC) Wessex University of Southampton Southampton UK

**Keywords:** collective efficacy, long‐term conditions, meta‐synthesis, self‐efficacy, self‐management

## Abstract

Social networks have been found to have a valuable role in supporting the management of long‐term conditions. However, the focus on the quality and how well self‐management interventions work focus on individualised behavioural outcomes such as self‐efficacy and there is a need for understanding that focuses on the role of wider collective processes in self‐management support. Collective efficacy presents a potentially useful candidate concept in the development and understanding of self‐management support interventions. To date it has mainly been utilised in the context of organisations and neighbourhoods related to social phenomena such as community cohesion. Drawing on Bandura's original theorisation this meta‐synthesis explores how studies of collective efficacy might illuminate collective elements operating within the personal communities of people with long‐term conditions. A qualitative meta‐synthesis was undertaken. Studies published between 1998 and 2018 that examined collective efficacy in relation to health and well‐being using qualitative and mixed methods was eligible for inclusion. Timing of engagement with others, building trust in the group, and legitimising ongoing engagement with the group arised as central elements of collective efficacy. The two themes forming third order constructs were related to the presence of *continuous interaction* and *ongoing relational work* between members of the group. Collective efficacy can develop and be sustained over time in a range of situations where individuals may not have intense relationships with one another and have limited commitment and contact with one another. Extending this to the personal communities of people with long‐term conditions it may be the case that collective efficacy enables a number of engagement opportunities which can be oriented towards assisting with support from networks over a sustained length of time. This may include negotiating acceptable connections to resources and activities which in turn may help change existing practice in ways that improve long‐term condition management.

**What is known about the topic**

- Social networks, reciprocity and relationality play a key role in the management of long‐term conditions in people's everyday lives.
- There is need to better understand how people engage with social capital, support and resources in a way that is acceptable and meaningful to them and members of their networks when managing and living with a long‐term condition.
- Collective efficacy is closely linked conceptually to the notion of self‐efficacy, but while self‐efficacy is widely used to assess and develop interventions supporting people with long‐term conditions collective efficacy is rarely used in such contexts.

**What this paper adds**

- Collective efficacy is a key concept illuminating the process of engaging and sustaining self‐management support in everyday settings.
- The paper illuminates the potential that the notion of collective efficacy can play in improved understanding of the interdependencies between the social and psychological processes involved in the everyday management of long‐term conditions.
- The findings might assist with identifying the ways in which people living with long‐term condition might engage network members in mobilising and sustaining support in domestic and community settings.

## 1 BACKGROUND

Historically the literature on long‐term condition management has emphasised the pivotal role played by individual knowledge, skills, motivation and capacity to self‐manage. There is growing recognition that self‐management is not an individual process, but one that depends on the support provided by members of one's social network. The involvement of network members in illness management forms an aspect of a collective network process, effort and change placing emphasis on collective agency rather than individual *self‐efficacy.* Recent findings highlight how social involvement with a diverse range of people, activities and groups provides social and psychological opportunities and resources which contribute to self‐management support (SMS) and physical and mental well‐being (Koetsenruijter et al., 2015; Reeves et al., 2014). This more recent focus has highlighted a need to better understand how people engage with social capital, support and resources in a way that is acceptable and meaningful to them and members of their networks when managing and living with a long‐term condition. Here, we consider networks as personal communities that extend beyond patient and carer dyads to include a larger group of a community of interconnected individuals (such as friends and colleagues) that are strongly or loosely connected with one another [referred to as a network of networks (Vassilev et al., 2011)].The dynamic of an individual operating within this type of networks may involve making decisions about when and who to contact, identifying and utilising resources that were previously underused, selecting some individuals over others, and providing a rationale that helps keep existing relationships content (Kennedy et al., 2015) This points to the need to explore the relationship between social capital and social support through paying greater attention to the process of engagement within social networks and the varying levels of collective efficacy of different networks, that is, their individual and collective capacity for such engagement (Vassilev, Rogers, Kennedy, & Koetsenruijter, 2014).

Collective efficacy was first mooted by Bandura (1986) who defined it as ‘a group's shared belief in its conjoint capabilities to organize and execute the courses of action required to produce given levels of attainments’. Referencing social cognitive theory, collective efficacy encompasses co‐ordinated, interactive and shared effort, beliefs, influence, perseverance and objectives in the pursuit of behavioural outcomes (Bandura & Kazdin, 2000). Collective efficacy is an ‘emergent property’ of the group, rather than the sum of the self‐efficacy levels of individual group members, and is ‘the product of the interactive and co‐ordinative dynamics of its members’ (Bandura, 1998). In Bandura's original work CE is conceptualised as a group process that can operate on the micro, meso and macro levels, where it is of relevance for understanding the common concerns of families, social networks, institutions and national communities (Bandura, 1997). Collective efficacy has most frequently been examined on the meso level to study different communities of belonging (kin, ethnic, cultural, faith) and place (neighbourhoods), and organisations the boundaries of which are relatively well‐defined (community care settings, schools, businesses) (Bazant & Boulay, 2007; Goddard, Burns, & Catty, 2004; Lawrence & Schiegelone, 2002; Sampson, Raudenbush, & Earls, 1997). When applied to neighbourhood safety for example collective efficacy is proposed to operate with reference to group support, motivation, responsibility, and the capacity to achieve common goals and willingness to intervene for the ‘common good’ (Sampson et al., 1997). Social cohesion and informal social control are identified as two major elements of CE in neighbourhoods: the former implicates solidarity and mutual trust, while informal social control is needed to fulfil the group expectation to be able to take action together. The notion of CE has been developed little beyond such applications on the meso level but the idea re‐emerged within studies of LTCM, where it has different nuances.

In relation to living with a LTC strategies and practices for management operate on the micro level and are likely to incorporate multiple and changing objectives, and to spread across a wide set of relationships and groups that individuals belong to (e.g. place of work, locality) (Vassilev et al., 2014). Given the specific complexities of relations within the personal communities of people experiencing a range of conditions there is a need to explore more fully the possible feasibility, utility and applicability of conceptualisations of CE to LTCM network support and design of complex interventions. This meta‐synthesis was designed to explore the existing conceptualisations and empirical research on CE as a means of illuminating the collective processes operating within the personal communities of people with LTCs, and identifying some of the conditions required for building and sustaining CE in such contexts.

## 2 METHODS

We used meta‐synthesis in order to identify the processes associated with developing and sustaining collective efficacy across different groups and to illuminate the relevance of these processes for the everyday management of long‐term conditions. We included a broad public health focus which included the more distal social elements and environment which are likely to impact on interactions in a local community which impact indirectly on health. Meta‐synthesis is an inductive method for synthesising and re‐interpreting in novel ways empirical data and conceptualisations across qualitative studies (Noblit & Hare, 1988). The method allows for the in‐depth understanding of the phenomena studied and its articulations across different contexts by exploring it on three levels. First order constructs are the narratives, experiences and interpretations of respondents. Second order constructs are the constructs and interpretations of the authors of the reviewed papers. Third order constructs are the interpretations, concepts and theories that illuminate the relationship between second order constructs and the research questions (Britten et al., 2002; Campbell et al., 2003; Noblit & Hare, 1988).

### 2.1 Search procedure

The literature search was conducted in January 2018 using the following key words: ‘collective efficacy’; ‘qualitative’; ‘focus group*’; ‘interview’; and ‘ethnograph*’. The following databases were searched: Web of Science; PubMed, Embase, Medline and the Cochrane Library. Additional studies were sought through manually checking references recorded in relevant studies.

### 2.2 Study selection

**Figure 1:**
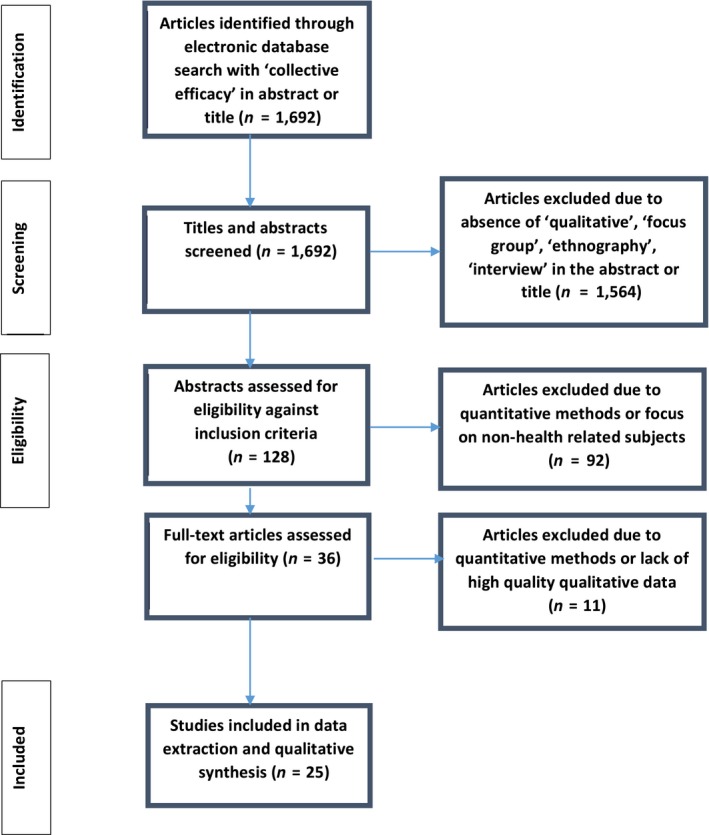
Process of paper selection

Inclusion criteria required that studies were: written in English; published 1998–2018; found to include the term ‘collective efficacy’; predominately qualitative or mixed methods; socio‐environmental factors that contribute to health and community and locality relevance. Exclusion criteria eliminated studies that were: predominately quantitative; non‐health related (e.g. education‐based, activism‐based, elite sports related). Studies were first identified against by a researcher (EJ): the initial search found 1,692 articles that contained ‘collective efficacy’ in the abstract, which was reduced to 128 articles using qualitative methods of analysis. These 128 articles were screened jointly by three reviewers (EJ, AK, IV) to assess eligibility. Papers were initially screened by title and abstract but if insufficient we also looked at the whole paper. We used the Blaxter (1996) guidance for evaluating qualitative papers. This included evaluation of the process of data sampling and generating themes, systematic presentation of findings that are credible and appropriate, discussion plausibly linking data interpretation and theory. Any discrepancies at this stage were discussed by the full team (including AR, RB). Feedback was collated in order to reach consensus, with 25 articles agreed for inclusion. The search and selection process is illustrated in Figure 1. Most commonly, studies examined collective efficacy in neighbourhood environments (*n* = 12) and public housing (*n* = 2). Other contexts included community organisations or groups (*n* = 4), within personal communities such as families and dyads, (*n* = 5) and in occupational settings (*n* = 2).

### 2.3 Data extraction and synthesis

**Figure 2:**
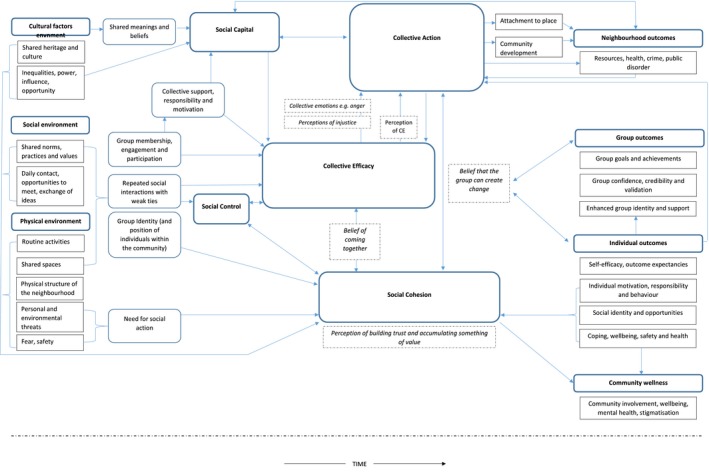
Concepts map

Given the limited use of the notion of collective efficacy in relation to personal communities and the management of long‐term conditions we started the review process by identifying conceptualisations of collective efficacy and the contexts within which they were discussed. Applied realist approaches informed the early stages of the review in conceptualising the potentially relevant mechanisms (Table 1 and Figure 2), and framing the key questions (Decoteau, 2017; Pawson, Tilley, & Tilley, 1997; Porter, 1993). Data from each of the 25 papers included in the final synthesis were each extracted by two team members. Using dedicated forms for standardising extraction, key findings and themes identified in the study were extracted. The data extraction form included details of the study setting and locality, sampling data collection and analysis. The key findings and themes identified as first order constructs. Author references to the notion of CE in terms of implications and policy recommendations were identified as second order constructs (e.g. ‘Social cohesion among neighbours combined with their willingness to intervene on behalf of the common good’ Sampson et al., 1997). The evidence was synthesised by a reviewer (RB) and findings were discussed within the team. A synthesis table was generated which included the social and environmental contexts, what were the conditions, circumstances, perceived barriers and facilitators associated with CE. A summary of the data is provided in Table 1. Visual representations of the relationships between constructs were developed (Figure 2); this process went through several iterations which were discussed by the team at each stage. Critical interpretations by reviewers of the necessary conditions for the development and sustainability over time of CE were developed as third order constructs.

**Table 1:** Summary of key finding of papers included within the review

Study	Country	Setting	Environmental contexts and social factors relevant for CE	Properties of collective efficacy	Mechanisms of impact (CE on outcomes)
Altschuler et al. (2004)	USA	Neighbourhoods	Availability of natural, open spaces; access to local services and resources influenced by sociodemographic factors (such food stores). Neighbourhood safety and threats result in CE through increased social capital and cohesion. Close local proximity of people within the neighbourhood drive CE.	Feelings of trust and reciprocal behaviour, associated with accumulating something of value.	CE is understood as the mechanism of collective change, translating social action in to neighbourhood resources, amenities and health outcomes. CE and social cohesion interact so that community involvement builds ties, which increased support and esteem.
Carter, Parker, and Zaykowski (2017)	USA	Neighbourhoods	Presence of ‘old heads’ who are able and willing to engage with local residents and with the full range of formal and informal groups and institutions in addressing disagreements and conflicts.	Trust invested in individuals to develop and negotiate acceptable solutions to concrete problems as and when they arise. Trust sustained if brokered across all relevant contexts.	CE is sustained through old heads who engage in, and build up, their communities through linking people to resources, solving problems, and establishing social ties.
Gerell (2015)	Sweden	Neighbourhoods	Repeated, everyday interactions with people in shared spaces such as stairwells and gardens promote increased CE. CE is influenced by routine daily activities and norms (such as saying hello). Lower CE associated with higher levels of social housing.	Shared mutual trust and willingness to intervene to address issues for the social good.	CE is a social mechanism that translates a strong sense of social cohesion into action. Through exerting control over the environment, residents were able to achieve CE despite social threats (of gang violence).
Kleinhans and Bolt (2014)	Holland	Neighbourhoods	Neighbourhood disorder and negative experiences increase fear and caution, lack of trust, lower perceived social control and willingness to act. Social interactions among neighbours (weak ties) result in feelings of familiarity; this is important for shaping collective perceptions of social cohesion, trust in the informal social control, capacity and willingness to act, shared norms, values and practices with other residents.	Social cohesion among neighbours combined with their willingness to intervene on behalf of the common good.	CE is a mechanism through which neighbourhoods can target perceived disorder. Positive everyday interactions (and resulting familiarity and respect for privacy) combined with perceived adherence to social norms result in willingness and resources to enact social control. Negative experiences (even perceived) override the positive effects of CE.
McNamara et al. (2013)	Ireland	Neighbourhoods	Stigmatised neighbourhood identity leads to adverse environments, characterised by division, lack of cohesion, action and engagement (particularly if this would result in exposure of stigmatised identity). A strong community identity and the belief that the group could cope with adversity were beneficial.	A groups’ ability to overcome adversity (where there is disadvantage).	CE is proposed to impact on action via a problem‐focused pathway and an emotion‐focused pathway, where collective emotions (i.e. anger) translate in to action. However, stigmatised identity had a negative influence on collective action, despite significant threats.
Moore et al. (2004)	USA	Neighbourhoods	Social inequalities and disparities impact on influence and ability to accumulate resources needed for CE. Views about the community (and the position of members within the community) result in vulnerabilities for those who are marginalised or socially excluded.	Mutual trust, reciprocity and willingness to act towards the common good in the neighbourhood interact to form CE.	CE is a moderating social variable which is temporary and dependent on the timing of events (i.e. may be high levels and experiences of CE immediately following a disaster but this interacts with other experiences of support over time).
Pegram et al. (2016)	USA	Neighbourhoods/community groups	Network of ministers in deprived areas developing strategic coalitions to manage violence. The network can mobilise other churches, community organisations, and link up with formal institutions.	Ability to engage with different community members, leaders, and organisations and work together towards reducing violence.	CE works through day to day interaction with local people, building familiarity between residents, stimulating informal social control, shaping and representing community views on youth violence, connecting high‐risk youth and their families to social services, providing a strong moral voice on violence in partnership with government entities.
Petrosino and Pace (2015)	USA	Neighbourhoods	Strong group identify (including mutual values, goals, interests, common culture, heritage and trust among members) is important for the development of CE. A common external threat (such as violence) is a precondition to CE (although it negatively undermined CE due to lower self‐esteem, lower perceived safety and threatened social identity).	The capacity to act, identified by intended or planned activities within the community. It represents the desire for a group to work together for the common good of the neighbourhood.	CE impacts on neighbourhood outcomes through the formation of a task force, visible leadership, and engagement of people (which was lacking in this community, so there was a lack of collective efficacy despite social cohesion).
Rogers, Huxley, Evans and Gately, (2008)	UK	Neighbourhoods	Neighbourhoods define individuals’ sense of identity, aspirations, opportunities, self‐esteem linked to well‐being and life satisfaction (the locality was a mediating influence which either increased a sense of personal vulnerability or resilience). Low social capital, ‘weak’ ties and lack of community organisation related to lack of CE development. People were reluctant to engage in collective activities, instead having a sense of self‐reliance and keeping ‘oneself to oneself’.		The combined resources of individuals (e.g. trust, norms, and social networks) and their integration into collective activity (e.g. sporting, religious, educational, cultural and artistic events and routines) linked with changes in outcomes. Low levels of community social capital are maintained by individual avoidant coping and enforced social isolation, with antisocial behaviour and lack of jobs negatively affecting mental well‐being.
Sargeant, Wickes, and Mazerolle (2013)	Australia	Neighbourhoods	CE varies according to neighbourhood characteristics. Community trust and willingness to engage in informal social control are related with less crime (despite disadvantaged characteristics). Police legitimacy (“trust” and the “obligation to obey” police) influences a generalised sense of trust in communities and in the development of shared norms for intervention.	‘Shared expectations for social action’ which can vary from informal intervention to formal controls.	CE proposed to mediate the relationship between neighbourhood structural characteristics and violent crime. Tensions between communities and institutional bodies (particularly those viewed as unjust, illegitimate or ineffective) result in low CE even if targeted, as individuals lack the skills to negotiate these relationships.
Turney, Kissane, and Edin (2013)	USA	Neighbourhoods	The physical and social characteristics of neighbourhoods and housing have important implications for quality of life. Crime and violence lead to reduced interactions as individuals keep to themselves. Feeling safe and sense of community (i.e. saying hi, co‐operating, and looking out for neighbours’ children) increased the sense of CE, greater efficacy and well‐being.	Social cohesion among neighbours and a willingness to work for common values in their low‐poverty neighbourhoods.	Collective efficacy fostered a sense of community co‐operation among neighbours, increased self‐efficacy, help people feel ‘more settled’, and helped support parenting.
Wickes (2010)	Australia	Neighbourhoods	Shared common interests and values, wealth, education were important for developing trust and expectations for control, interpersonal connections and relationships with other people.	A shared belief in the groups’ capabilities to organise and execute the courses of action required to produce given levels of attainments.	Important symbols and characteristics of assumed (affluent) identity established trust and shared values, such as removing the visual effects of crime.
Freedman, Pitner, Powers, and Anderson ( 2012)	USA	Housing	A strong attachment to place was associated with a sense of neighbourhood pride and ownership. Shared (African American) culture and heritage were important for a sense of cohesion. A belief that the community would come together and the ability to create change facilitated CE. A sense of powerlessness, feeling ‘stuck’ in public‐housing communities due to poverty, under‐education and few financial (or other) resources detracted from CE.	Social cohesion among neighbours combined with their willingness to intervene on behalf of the common good.	Collective efficacy facilitated community wellness when residents felt community members could work together to address community concerns, expressed a sense of agency for making community change and showed strong connection or unity among other residents based on shared African American heritage and cultural values.
Shin (2014)	USA	Housing	Physical proximity, everyday interactions, social support and shared activity settings (physical location) were important for the development of CE. Cultural background was also important (with cultural brokers acting on behalf of individuals and the residents as a whole).	Social cohesion and the willingness to intervene on behalf of the common good; defined in relation to a general support available whenever needed, a sense of belonging and responsibility for others.	CE was associated with various types of support, such as navigating the outside world, sharing transport, socialisation and emotional support (providing a sense of belonging and security), preserving esteem and independence. The cultural broker helped to mobilise resources and act as a bridge between US and Korean culture.
Teig et al. (2009)	USA	Community groups	The community group activities included volunteering and leadership. Neighbourhood engagement and recruitment worked as concrete mechanisms to support the development of CE. Leaders were reliable champions willing to take action; neighbourhood activities made the garden visible to others and recruitment activities broadened the range of social networks.	The link between mutual trust and a shared willingness to intervene for the common good of the neighbourhood—an expectation of action.	CE was a mediating effect between the community gardens and community health. The development of CE led to reciprocal relationships between volunteers, new social norms (which discouraged violence and crime), sharing of food and advice, social support, new social connections with the community and a willingness to help one another.
Mok and Martinson (2000)	Hong Kong	Community groups	Feelings of trust and belonging needed to develop before people were willing to engage, for mutual disclosure and role‐modelling. Barriers were cultural—not wanting an expectation of reciprocity.	A thematic outcome—coming together a collective power. This social action was very rare and not very desired—empowerment concern was at a personal level.	
Ingram et al. (2009)	USA	Community groups	A sense of self‐efficacy, social cohesion—related to clear expectations and commitment to the group, and social support—through the development of personal bonds and sharing of personal information.	The belief that the group can improve their lives through collective effort, made up of self‐efficacy, social support, and social cohesion.	Collective efficacy impacts on participants desire to walk/motivation, and attitudes to walking. Increased group collective efficacy builds individual self‐efficacy, social cohesion and development of personal bonds (so in a reciprocal cycle). CE also leads to the formation of group goals and increased knowledge regarding the benefit of walking (which leads to increased walking behaviour).
Fisher and Gosselink (2008)	USA	Community groups	Belief associated with goal attainment important for being able to utilise resources to achieve goals. Group membership and participation is important for developing group efficacy, which interacts with and impacts on individual efficacy& promotes group goal achievement and validates effort.	Collective organisation for social action.	CE increases individual motivation, success, sense of achievement and overall well‐being; promotes the development of friendships and sense of group power, increased group successes and sharing of ideas, and sustainability of the group.
Beverly and Wray (2008)	USA	Personal communities	Individual efficacy, motivation, responsibility, and outcome expectancies are important for individual behaviour. In dyads, observed partner behaviour and interactions among participants is important for determining collective support, collective motivation and collective responsibility. It requires a complex process of negotiation; getting it just right—tensions between illness and relationship work.	A shared perception of capability to successfully perform behaviour (an emergent process based on shared beliefs, the actions taken and collective effort towards desired outcomes).	Collective efficacy critically important in long‐term sustainability of behaviour through a process of dynamic reciprocity. Collective efficacy evolves from performance feedback and teamwork.
DeKeseredy, Rogness, and Schwartz (2008)	USA	Personal communities	Isolation, rurality and reinforcement of existing conservative norms of violence and male dominance reinforced strong male bonding relationships. Weak ties insufficient in counteracting the negative trajectory sustained by strong ties. Power of strong ties strengthened or weakened depending on physical proximity.	A mutual trust among neighbours combined with a willingness to act on behalf of the common good. Comprises of informal social control and social cohesion and trust. Discussed negatively in this example (repressing women).	High levels of CE threaten women seeking freedom from divorce and sexual assault. Women fear strong social ties between men who sexually/ physically assault women. The fear of many women is exacerbated by knowing that the men who abuse them have strong social ties with male peers who sexually or physically assault women ‐ many rural men can also rely on their male friends and neighbours, including those who are police officers, to support a violent patriarchal status quo, which to them is acting on ‘behalf of the common good’.
Jarrett, Bahar, and Taylor (2011)	USA	Personal communities	Access to social (family and nonfamily adults) and institutional (recreational and physical activity) resources impacted on the level of youth physical activity. Age and education of caregiver impacted on negotiation of resources.	One of five social neighbourhood processes along with neighbourhood resource; collective socialisation; social control; epidemic.	Low CE was related to low collective socialisation, low social control and low resources, which negatively impacted on children's’ physical activity. Having safe spaces to play and collective supervision/ trust with neighbours increased activity.
Kennedy et al. (2015)	UK	Personal communities	Diverse (‘generative’) social networks which can generate support from lots of sources including weak ties were important for CE. One person was often the driver of collective efficacy (often a spouse) by building in the changes needed (such as diet) in to everyday life. Financial resources were a limiting factor in management of health.	Developing a shared perception and capacity aimed at successful management through shared efforts and objectives.	When collective efficacy was apparent it seemed to encourage or facilitate the undertaking of healthy lifestyle practices—which were more likely when this was reciprocal.
Vassilev et al. (2014)	UK	Personal communities		Developing a shared perception and capacity to successfully perform behaviour through shared effort, beliefs, influence, perseverance and objectives.	Three mechanisms linking social networks to health outcomes: 1. Network navigation (identifying and connecting with resources in the network i.e. who and when to contact) 2. Negotiating relationships (re: relationships, roles, expectations, means of engagement and communication between network members—to decide on responsibility, level and type of involvement). 3. Collective efficacy
Bess et al. (2009)	USA	Occupational	Surplus powerlessness is generated by fear of reprisal, role overload and (lack of) necessary capacity and skills (which are needed for participatory processes). Participation a continuum from non‐participation to political engagement.	Discussed as the opposite to surplus powerlessness	Trust and more equal power distribution; skills building around participatory processes; specific roles or structured ways; more time to teach these in communities with many social and health needs.
Howarth et al. (2012)	UK	Occupational	CE develops within a negotiated shared space, which supports the exchange of inter‐ professional ideas, opportunity to come together, expand relationships, daily contact. Linked to mutual/reciprocal respect among all team members and respect towards each other's competencies and abilities. Teams who understood individual roles and areas of expertise knew when they needed to refer to other teams.	A belief in the team that they will achieve their intended goals (i.e. level of confidence in their professional competence to perform successfully). CE matures over time as relationships develop.	A sense of team CE strengthened team confidence in itself as a team and professional credibility that ultimately supported partnership working with patients which the team believed had empowered patients to participate in care decision‐making.

## 3 RESULTS

The themes developed in the analysed papers, second order synthesis, were summarised within three overarching themes: the fragile nature and changing salience of collective efficacy over time, relationships and trust between group members, legitimising ongoing engagement with the group (see Table 2).

**Table 2:** Example concepts from second order extraction

Fragile sense and salience of collective efficacy	Individuals within the group prepared to give more effort and time to the collective under critical circumstances (Moore et al., 2004);
	Crime or other negative experiences that undermined personal self‐esteem, safety and identity detrimental to collective efficacy (Jarrett et al., 2011; Kleinhans & Bolt, 2014; McNamara et al., 2013; Turney et al., 2013);
	Deprived neighbourhoods which lack the necessary resources and capacity to organise action or mobilise available resources (Jarrett et al., 2011; Rogers et al., 2008; Wickes, 2010);
	Skills needed within members of personal community to allow good communication and negotiation. This takes time to have an effect (Pegram et al., 2016);
	Collective efficacy shaped by lack of clear visible and effective leadership (Carter et al., 2017; Pegram et al., 2016);
	Collective mobilisation requires availability of trusted people with skills for understanding and engaging with formal organisations where there is distrust in such organisations (Altschuler et al., 2004; Carter et al., 2017; Wickes, 2010);
	Engagement may be prevented where there is expectation of reciprocity and unwillingness or inability to reciprocate (Kennedy et al., 2015; Moore et al., 2004);
	A surge of support at times of health or other crisis situation which tends to decrease over time. Sustaining support is difficult and constant demands could lead to disillusionment and even anger in the personal community ‐ people with chronic illness wary of putting such burdens on others because of this (Beverly & Wray, 2008; Vassilev et al., 2014);
	Deprivation in particular can lead to feelings of being ‘stuck’ in the situation (Bess et al., 2009), powerlessness generated by fear and burden, disengagement from individuals, formal and informal institutions (Carter et al., 2017; Pegram et al., 2016);
	Disengagement particularly relevant to marginalised groups or individuals with stigmatised identities (McNamara et al., 2013);
	Coping with a constellation of factors associated with deprivation and stigma can prompt social isolation and avoidance (Wickes, 2010)
Relationships and trust between group members	CE emerges over time as a result of trust and relationship work among group members (Carter et al., 2017; Pegram et al., 2016; Vassilev et al., 2014);
	Need for opening space of negotiation; should not be taken for granted; engagement with neighbours requires familiarity (Pegram et al., 2016);
	Negotiation a long‐term process, but with a historically established pattern within relationships, localities, and networks (Kennedy et al., 2015). There are different styles of negotiation within groups and relationships: formalised/integrated into everyday life;
	Repeated interactions between neighbours in shared spaces build familiarity and confidence (Gerell 2015; Howarth et al., 2012; Kleinhans & Bolt, 2014);
	Sense of confidence shaped by routine daily routines, or routine activities, along a spectrum from greeting each other to collectively working together for improvement of their yard (Gerell 2015);
	Public familiarity can arise and be sustained by greeting each other on the street and (being able) to have a quick chat. Superficial contacts like these contribute greatly to recognizing others and becoming ‘intimate strangers’ or more (Turney et al., 2013);
	Collective efficacy developed by building personal relationships between some group members (Fisher & Gosselink, 2008; Ingram et al., 2009; Kennedy et al., 2015);
	Collective efficacy related to tacit knowledge and doing the right thing (Beverly & Wray, 2008; Kennedy et al., 2015);
	Confidence for engagement can be built through person mediated trust in unfamiliar or hostile others: through the involvement of trusted and respected people (Carter et al., 2017);
	Process of negotiation, such as creating a set of common goals between group members is a key component in the development of CE (Beverly & Wray, 2008; Ingram et al., 2009; Kennedy et al., 2015; Vassilev et al., 2014);
	Getting to know others helps people to work together, through familiarity (Pegram et al., 2016), understanding the skills and capabilities others have (Bess et al., 2009; Howarth et al., 2012) or in helping others to ‘bridge the gap’, when their own personal capabilities and resources are lacking (Shin, 2014);
	CE associated with sharing personal information, developing a sense of belonging and establishing feelings of trust between network members (Ingram et al., 2009; Mok & Martinson, 2000);
	As relationships develop, social support and norms are strengthened, building confidence and credibility of the group (Howarth et al., 2012; Teig et al., 2009)
	However, trust and strong relationships can also lead to informal social control with negative outcomes (DeKeseredy et al., 2008; Moore et al., 2004).
Legitimising ongoing engagement with the group	Work required through authoritative support in the form of visible leaders negotiating objectives across formal and informal institutions (Carter et al., 2017; Pegram et al., 2016);
	A set of group members driving collective goals and actions (Kennedy et al., 2015; Pegram et al., 2016; Petrosino & Pace, 2015; Shin, 2014; Teig et al., 2009);
	In order to facilitate collective efficacy, one has to actively pursue and maintain neighbourhood relationships and respect (Carter et al., 2017);
	Capacity to encourage social cohesion and trust while negotiating deeply rooted stereotypes, prejudices, and perspectives across group members and relevant institutions (Carter et al., 2017; Pegram et al., 2016; Wickes, 2010);
	Review of common goals is ongoing and important (Ingram et al., 2009; Kennedy et al., 2015);
	Feedback on the performance and achievements of the collective promote further action over time (Beverly & Wray, 2008; Fisher & Gosselink, 2008);
	Tensions between illness and relational work (Beverly & Wray, 2008; Kennedy et al., 2015);
	Power of strong ties not necessarily leading to CE, may be strengthened or weakened depending on physical proximity (DeKeseredy et al., 2008);
	Ongoing resources are also required to sustain collective efforts, which may be disproportionately affected by social disadvantage and inequalities (Moore et al., 2004; Rogers et al., 2008);
	CE can decrease where there is lack of resources even when there is ongoing need for collective action (Moore et al., 2004)

### 3.1 The fragile nature and changing salience of collective efficacy over time

In circumstances leading up to a critical event, such as disaster or immediate threat to the group, collective efficacy may emerge quickly, presenting as a ‘momentary upsurge in collective unity’ (Moore et al., 2004). It appears that, under critical circumstances, individuals within the group are prepared to give more effort and time to the collective generated by the sense of a common purpose than under more mundane circumstances. However, the presence of a threat alone is insufficient to bring about collective efficacy, with the evidence suggesting that interactions that occur between the environment and timing of action are pertinent (Petrosino & Pace, 2015). Several studies highlighted the fragility of collective efficacy and how it could be undermined by environmental influences. This included the exposure to the effects of crime or other negative experiences such as domestic violence that undermined personal self‐esteem and feelings of personal safety and identity (Jarrett et al., 2011; Kleinhans & Bolt, 2014; McNamara, Stevenson, & Muldoon, 2013; Pegram, Brunson, & Braga, 2016; Turney et al., 2013; Wickes, 2010). Perceived threats and experiences can outweigh the potential positive effects of collective efficacy (Bazant & Boulay, 2007). Compared to locations of affluence the generation of collective efficacy is seemingly more difficult in deprived neighbourhoods which lack the necessary resources and capacity to organise action or mobilise available resources (Altschuler, Somkin, & Adler, 2004; Bess, Prilleltensky, Perkins, & Collins, 2009; Jarrett et al., 2011; Moore et al., 2004; Rogers et al., 2008). This can lead to people feeling ‘stuck’ in the situation (Freedman et al., 2012) and powerless due to fear and burden (Bess et al., 2009), which have particular meaning to the social situations of marginalised groups or individuals with stigmatised identities (McNamara et al., 2013; Moore et al., 2004). Coping strategies to deal with the latter can bring about further social isolation and avoidance (Rogers et al., 2008). A lack of clear visible leadership around a matter of group or locality concern can also suppress collective efficacy (Pegram et al., 2016; Petrosino & Pace, 2015), as do lack of skills with engaging with, and mistrust of formal organisations (Rogers et al., 2008; Sargeant et al., 2013). Cultural barriers, such as expectations from others for reciprocity, may also contribute to a lack or diminution of collective efficacy (Mok & Martinson, 2000).

### 3.2 Relationships and trust between group members

In many cases, collective efficacy is generated over time as a result of trustworthiness, and the ongoing relationship among group members (Vassilev et al., 2014), is an outcome of repeated interactions between neighbours in a shared environment (Gerell 2015; Howarth, Warne, & Haigh, 2012; Kleinhans & Bolt, 2014) and often the result of dedicated attempts at building personal relationships between group members (Fisher & Gosselink, 2008; Ingram, Ruiz, Mayorga, & Rosales, 2009). The process of negotiating a set of common goals (such as starting walking or joining a walking group) between group members is a key component in the development of collective efficacy (Beverly & Wray, 2008; Ingram et al., 2009; Vassilev et al., 2014). Familiarity is another component: getting to know others helps people to work together, through building a sense of safety (Pegram et al., 2016), opportunity understanding, communicating and sharing mutual or exchangeable skills and capabilities (Bess et al., 2009; Howarth et al., 2012) or in helping others to ‘bridge the gap’, when their own personal capabilities and resources are lacking (Shin, 2014). Collective efficacy has been associated with the sharing of personal information, developing a sense of belonging and establishing feelings of trust and trustworthiness between network members (Ingram et al., 2009; Mok & Martinson, 2000). As relationships develop, social support and norms are strengthened, building confidence and credibility of the group (Howarth et al., 2012; Teig et al., 2009). Converse to this some studies highlight deviant cases, where trust and strong relationships between certain group members lead to informal social control with negative outcomes (DeKeseredy et al., 2008). In the example of domestic violence strong relationships and high levels of mutual trust between men led to a high degree of collective efficacy for reinforcing established social norms (of male dominance and violence in rural communities), making it difficult for women victims to resist or tackle the problem (DeKeseredy et al., 2008).

### 3.3 Legitimising ongoing engagement with the group

Following the emergence of collective efficacy, whether triggered by a critical incident, or nurtured through the development of relationships over time, the evidence reviewed suggests that further work must be undertaken to sustain the process. This involves the deployment of authoritative support in the form of visible leaders or a set of group members responding to individual concerns, and driving collective goals and actions (Carter et al., 2017; Pegram et al., 2016; Petrosino & Pace, 2015; Shin, 2014; Teig et al., 2009). Constancy of effort and ongoing review of common goals are relevant to this (Ingram et al., 2009) as is feedback on the performance and achievements of the collective which promotes and reinforces action over time through increased self‐ and collective‐efficacy (Beverly & Wray, 2008; Fisher & Gosselink, 2008). Such a process is enhanced through the presence of trusted individuals who are engaged on a day‐to‐day basis with different members of the group, make themselves available and are able to formulate, respond to, and negotiate individual and group concerns as they arise (Carter, 2017; Pegram et al., 2016). The ability to access and build in new resources are also required to sustain collective effort, the extent to which this is possible is influenced by social disadvantage and inequalities (Moore et al., 2004; Pegram et al., 2016; Rogers et al., 2008) meaning that collective efficacy decreases even when there is a clear ongoing need and perceived advantage for collective action (Moore et al., 2004).

### 3.4 Collective efficacy in the context of long‐term condition management: third order synthesis

**Figure 3:**
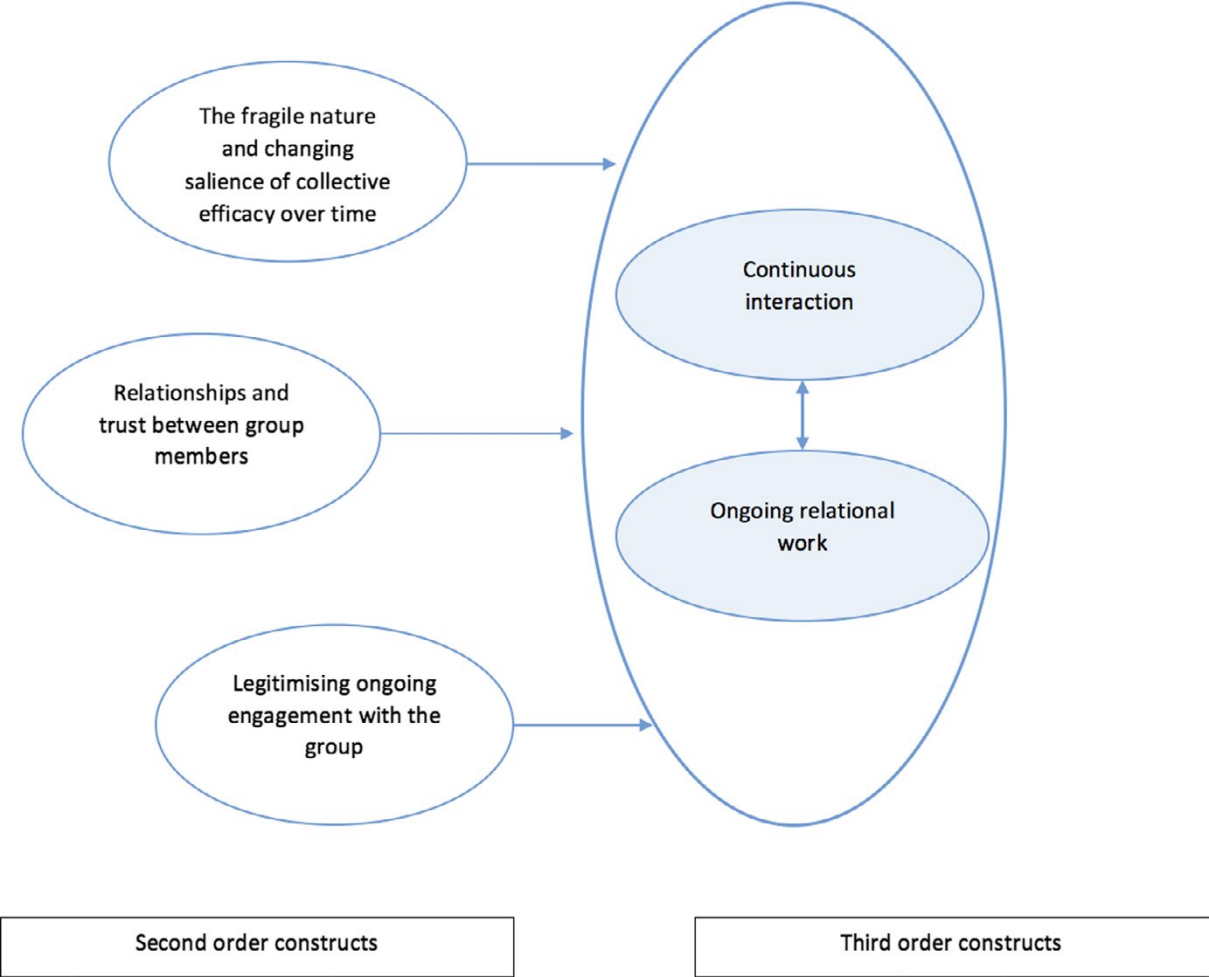
Second and third order constructs

The summary of themes and observations by authors of the original papers were reinterpreted in relation to two key questions we posed at the beginning of the review: what are the conditions required for building and sustaining CE?, and how can this knowledge inform our understanding of the collective processes operating, on the micro level, within the personal communities of people with LTCs?. The third order synthesis indicates that *continuous interaction* and *ongoing relational work* is the necessary conditions for the development and sustainability of CE (see Figure 3). Specifically, the presence of *continuous interaction* between (not necessarily all) members of groups is associated with the development of familiarity, a sense of association, and weak forms of trust and trustworthiness. The reviewed studies indicate that the presence of shared activities (cultural, sporting, recreational, professional, religious, artistic, educational), the use of collective spaces (gardens, stairwells and communal areas) as part of routine daily activities, and opportunities for minimal daily interaction, expressions of friendliness, indications of concern for members of the group or the group as a whole, which may be sufficient to generate the required collective efficacy needed to promote well‐being or leverage social involvement and inter‐personal support. Collective efficacy also requires a degree of *ongoing relational work* where individuals have the opportunity and capability to negotiate acceptable ways of identifying and working towards achieving objectives of individual or mutual value. These may include things such as improving access to food stores, green spaces, recreational activities, or housing, removing visual effects of crime, addressing concerns about social exclusion, safety, or organisational performance, negotiating perceived or real cultural differences. These findings are consistent with Bandura's original theorisation where CE is understood as an emergent property of relationships between members of groups (Bandura, 1986; Sampson et al., 1997) orientated towards addressing individual and collective concerns. Indeed, the structural characteristics of groups such as a sense of common identity and belonging to the group; homogeneity of background and experiences of group members; the presence of well‐defined and shared beliefs and expectations (Sargeant et al., 2013; Wickes, 2010); capacity of the group to act as a single unit (Beverly & Wray, 2008; Fisher & Gosselink, 2008; Kleinhans & Bolt, 2014; Petrosino & Pace, 2015; Teig et al., 2009), while relevant, were seemingly neither necessary nor sufficient conditions for developing and sustaining CE (see Table 3). Our findings show that CE can operate in small and large‐scale groups, where there might be multiple and conflicting articulations of the common good, and where a single common goal, that is well‐defined and widely shared across the whole group, might be absent. Identifying continuous interaction and relational work as the necessary conditions for CE suggests that relationality in CE needs to be understood broadly in terms of both process and outcome, that is, as the group capacity to mobilise towards identifying, negotiating and doing the right thing at the right time while adapting to changing contexts (rather the narrower definition of achieving a fairly well defined common goal).

**Table 3:** Necessary conditions for developing and sustaining collective efficacy in personal communities

Necessary and sufficient structural conditions and relational processes	Relevant, but not necessary or sufficient structural conditions
Sense of association between some members of the group	Strong sense of group identity and sense of belonging
Weak ties and familiarity among some members of the group/network	Strong ties of commitment and trust among all/most members of the group
Heterogeneity of group members	Homogeneity of group
Loose group boundaries, multiple overlapping sub‐networks	Clearly defined group boundaries
Capacity for articulation and mobilisation across multiple objectives changing over time and adapting to contexts	Capacity for mobilisation in relation to a clearly defined objective
Continuous interaction between some members of the group	
Ongoing relational work where members of the group can negotiate objectives orientated to some common goal or good and ways of achieving them	

This metasynthesis extends earlier applications of the notion of CE to neighbourhoods and organisations (Goddard et al., 2004; Sampson et al., 1997) by indicating that that the notion can be applied at a micro level with reference to the personal communities of people with LTCs. In contrast to organisationally based groups of people, where group membership, boundaries and objectives are framed by institutional logics (e.g. school or student performance, competitiveness, innovativeness, etc.) and thus relatively closed for group deliberation, personal communities operate in the open systems of people's everyday life where objectives, concerns and social roles are more likely to vary and fluid. Personal communities are constituted of relationships which include a range of expectations, responsibilities and forms of reciprocity (e.g. towards and between close and distant family members, friends, neighbours, acquaintances, colleagues, healthcare professionals, etc.). Additionally, the objectives and priorities of people with LTCs are multiple, competing and change over time. These may include managing the experience of symptoms in different everyday situations, concern for the well‐being of other network members, ability to reciprocate and feel valued by others, capabilities to adopting changes orientated to ameliorate or lessen the impact of living life with a particular LTC, building individual and network connections over time, and negotiating and achieving a range of activities that are valued as part of everyday life. Thus, the individual and collective capacity of network members to do relational work in negotiating and navigating relationships across the multiple contexts of LTCM is a key aspect of collective efficacy when mobilising and sustaining self‐management support. Specifically, the everyday LTCM necessitates making judgements as to which existing relationships are required or need adjustment and which new ones need to be developed in undertaking self‐management support (Cramm & Nieboer, 2014; Pilgrim, Rogers, & Bentall, 2009; Vassilev et al., 2013). Approaching network members for help involves considerations as to how one might fulfil the expectations of reciprocity and acceptability of receiving support from others and how to resolve inherent tensions. For example, the desire for independence and autonomy may take precedence over the need for physical assistance, and act as a reason for not activating, or navigating support even when it is available. The preserving of a pre‐existing identity and roles in relation to specific close family ties (such as a son or daughter) or distrust and untrustworthiness of formal institutions may also preclude or promote the seeking and harnessing of support (Kennedy et al., 2015; Moore & McArthur, 2007).

Understanding collective efficacy as a process and of relationships helps to illuminate the roles and make—up of groups (e.g. size, homogeneity, density, fragmentation) and types of ties (e.g. relationships perceived to be strong bonding or weak bridging) might have for mobilising and sustaining support. Thus, while homogeneity has been identified as relevant to mobilisation of support in neighbourhoods and organisations, heterogeneity and diversity of personal communities are related with capacity for LTCM. Specifically, people whose personal communities include a range and diversity of relationships such as friends, pets, neighbours and activity groups, (including where many network members do not know each other) report better self‐management support and well‐being than people whose personal communities primarily consisting of family members (Litwin, 1998; Reeves et al., 2014; Vassilev et al., 2016). This may in part be due to opportunities for improve access to information and personal experiences available within a wider network which also may account for a reduction of burden on intimate relationships and potential to access support that is easier to reciprocate and less consequential if it fails to materialise (than is the case with intimate others) and enable access to activities that allow people to feel valued for their skill and competence outside their roles within families (Rogers et al., 2014).

## 4 CONCLUSIONS

Interest in collective efficacy has traditionally been through policy concerns about local risks to safety and deprivation of neighbourhoods and communities, and institutional underperformance. This focus tends to predefine collectives of interest as tight‐knit communities and to associate collective efficacy with the structure and properties of these. A narrow definition of CE is less applicable when CE is viewed in the context of LTCM, located in personal communities that are likely to resemble more loosely inter‐connected networks than tight‐knit communities. Despite the close link between notions of self‐efficacy and collective efficacy in Bandura's work it is only self‐efficacy that has achieved wide use and recognition, which may in part be reflection of the narrow interpretation of the notion of collective efficacy and the poor fit of such an interpretation with the openness of the social networks and everyday settings within which LTCM is located.

This meta‐synthesis found that the necessary and sufficient conditions for building CE were continuous interaction and ongoing relational work within groups where group members can be linked through weak ties of acquaintanceship and familiarity (Pegram et al., 2016; Pilgrim et al., 2009). These findings are consistent with the original theorisation of CE and enable its application on the micro level in relation to personal communities. Specifically, ongoing relational work within groups where group members negotiate how to achieve a common objective, what the objective is and how to make it acceptable to different group members is likely to be relevant for all groups, but is of central importance on the micro level in negotiating the tensions inherent in utilising existing resources and mobilising social support (Ray & Street, 2005; Waverijn, Heijmans, Spreeuwenberg, & Groenewegen, 2016).

Bandura's concept has stood the test of time but elaborating it in relation to personal communities makes it relevant to LTCM. The notion of CE offers a link between individual level self‐management processes, such as self‐efficacy, and access to and capacity to mobilise social capital and environmental resources when living with a LTC (Foss et al., 2016). Specifically, improvements in CE for people with LTCs can be sought where there is low intensity but wide‐ranging support for developing meaningful engagement opportunities dispersed within personal communities (e.g. in engagements with health professionals, in neighbourhoods, places of work, leisure, worship, education). This may include opportunities to improving individual and collective understanding of illness experiences; but also the ability to situate this understanding in relation to concrete concerns, valued activities and identities within personal communities, and the capacity of individuals and members of their personal communities to change existing practice in ways that improves LTCM while enhancing individual and collective well‐being (Entwistle & Watt, 2013).

Further exploration and theorisation of such processes is promising to contribute to developing a social network approach to SMS and lead to an improved understanding of the interdependencies between the social and psychological processes involved in the everyday management of a LTC. This is particularly relevant as many linked policy interventions designed to support LTCM, such as social prescribing for example, show deficits in understanding of the environments and interactions involved and resources needed. Further research might also aim to identify the mechanisms and structural characteristics associated with collective efficacy within and across the neighbourhoods, workplaces, and personal communities of people with LTCs, and explore the development of tools with which to measure collective efficacy across different contexts.

## CONFLICT OF INTEREST

We declare no conflict of interest.
